# Immunotherapy With Interferon α11, But Not Interferon Beta, Controls Persistent Retroviral Infection

**DOI:** 10.3389/fimmu.2021.809774

**Published:** 2022-01-20

**Authors:** Mara Schwerdtfeger, Julia Dickow, Yasmin Schmitz, Sandra Francois, Zehra Karakoese, Anna Malyshkina, Torben Knuschke, Ulf Dittmer, Kathrin Sutter

**Affiliations:** ^1^ Institute for Virology, University of Duisburg-Essen, Essen, Germany; ^2^ Institute for Translational HIV Research, University of Duisburg-Essen, Essen, Germany; ^3^ Institute for Medical Microbiology, University Hospital Essen, University of Duisburg-Essen, Essen, Germany

**Keywords:** Type I IFNs, retroviral infection, Friend retrovirus, persistent infection, immunotherapy, cytotoxic CD8^+^ T cells

## Abstract

Type I Interferons (IFNs), including numerous IFNα subtypes and IFNβ, are key molecules during innate and adaptive immune responses against viral infections. These cytokines exert various non-redundant biological activities, although binding to the same receptor. Persistent viral infections are often characterized by increased IFN signatures implicating a potential role of type I IFNs in disease pathogenesis. Using the well-established Friend retrovirus (FV) mouse model, we compared the therapeutic efficacy of IFNα11 and IFNβ in acute and chronic retroviral infection. We observed a strong antiviral activity of both IFNs during acute FV infection, whereas only IFNα11 and not IFNβ could also control persistent FV infection. The therapeutic treatment with IFNα11 induced the expression of antiviral IFN-stimulated genes (ISG) and improved cytotoxic T cell responses. Finally, dysfunctional CD8^+^ T cells solely regained cytotoxicity after IFNα11 treatment. Our data provide evidence for opposing activities of type I IFNs during chronic retroviral infections. IFNβ was shown to be involved in immune dysfunction in chronic infections, whereas IFNα11 had a strong antiviral potential and reactivated exhausted T cells during persistent retroviral infection. In contrast, during acute infection, both type I IFNs were able to efficiently suppress FV replication.

## Introduction

Type I IFNs belong to a multigene family consisting of numerous IFNα subtypes but only one IFNβ, IFNϵ, IFNκ, and IFNζ/limitin (1). All IFNα subtypes have similarities in structure, like the lack of introns or the length of the protein (161-167 amino acids), and their protein sequence is highly conserved (75 – 99% amino acid sequence identity) (2, 3). Interestingly, they all bind to the same ubiquitously expressed IFNα/β receptor (IFNAR), but their biological activities differ (4). Binding to the receptor leads to the activation of the classical Jak-STAT-signalling cascade, however also other signalling pathways become activated upon type I IFN binding [reviewed in (5)]. As a consequence, numerous and partly subtype-specific ISGs are transcribed with direct antiviral, immunomodulatory, but also regulatory properties.

IFNα2 is used in clinical applications since 1983. Interestingly, it is still the only subtype used for IFNα therapy to date. In contrast to most antiviral drugs, IFNα does not only prevent viral infection of cells, but it is also able to eliminate virus from host cells. For many years, IFNα2 has been used as the standard therapy for hepatitis C virus (HCV) infections alone or in combination with other antiviral drugs. Due to the development of more effective therapies with direct-acting antivirals, IFN-based therapy against chronic hepatitis C was no longer recommended in 2016. However, IFNα2 is still the standard treatment option for chronic hepatitis B patients, but only about 30% of patients respond to the therapy, of which only a few patients show complete viral clearance (6). IFNα2 has well-known adverse effects, which lead to discontinuance in approximately 15% of patients. Many clinical trials have analyzed the therapeutic potency of IFNα2 as monotherapy or in combination with antiretroviral therapy against HIV (7–9), but the therapeutic outcome was disappointing. We could recently show, using HIV-infected PBMCs and LPMCs as well as in HIV-infected humanized BLT mice, that other IFNα subtypes are much more potent in restricting HIV replication than IFNα2 (10, 11). Combination therapy of antiretroviral drugs together with IFNα14 further reduced the viral loads in chronically HIV-infected humanized mice (12), suggesting that IFN therapy with the right subtype (increased antiviral and immunomodulatory activity, reduced side effects) might still be an option to treat HIV infection. We found that IFNα14 reduced viral loads and improved NK cell responses in acutely HIV-infected humanized BLT mice with no sign of T cell hyperactivation or dysfunction (10). Furthermore, IFNα14 treatment during established HIV infection of humanized BLT mice in combination with antiretroviral treatment further reduced viral loads (12). However, for chronic HIV infection type I IFN induced hyperimmune activation is still controversially discussed (9, 13–18). Several studies showed that a type I IFN signature in chronically HIV-infected humanized mice was associated with T cell dysfunction and a lack of immune control of the virus (19–21). They suggested that IFN therapy might be detrimental during chronic HIV infection, but they did not distinguish between IFNβ and IFNα responses. Previous reports on chronic lymphocytic choriomeningitis virus (LCMV) infection of mice confirmed an antiviral role of type I IFNs during acute infection, but a rather detrimental role during chronic LCMV infection (22, 23). They reported that type I IFNs initiate an immunosuppressive program that represses antiviral immunity and facilitates persistent LCMV infection. However, the same authors showed some years later, that only IFNβ and not IFNα impaired antiviral immunity and supported persistent LCMV infection (24). Thus, IFNα might still be an option for the treatment of chronic infections, including HIV.

Using the well-established Friend retrovirus (FV) mouse model, we aimed to analyze the therapeutic potential of different type I IFNs during acute and chronic retroviral infections. The FV complex is comprised of two retroviruses: the replication-competent helper virus called Friend murine leukemia virus (F-MuLV), which is non-pathogenic in adult mice, and the replication-defective, pathogenic spleen focus-forming virus (SFFV) (25). FV induces erythroleukemia in susceptible mice. In contrast, resistant strains, such as the C57BL/6 mice that were used in the current study, mount potent immune responses during acute infection and recover from disease (26), but the viral control is incomplete leading to a life-long chronic FV infection. In the present study we addressed the distinct and non-redundant roles of IFNα and IFNβ during acute and chronic retroviral infection. Murine IFNα11, which was previously shown to efficiently control acute FV infection and improve NK cell effector functions (27), was selected from the IFNα subtype family for this comparison. Interestingly, therapeutic application of IFNα and IFNβ was effective in controlling acute FV infection, but only IFNα suppressed viral replication during chronic FV infection.

## Material and Methods

### Mice, Peptide and Virus

Female C57BL/6 mice were purchased from Envigo. All mice used for experiments were at least 8 weeks of age and were followed by the ARRIVE guidelines and maintained in accordance with the regulations and guidelines of the institutional animal care and use committee of the University of Duisburg-Essen, Germany. Peptide derived from the FV Gag protein (sequences: CCLCLTVFL) (28) was used for the *in vivo* cytotoxicity assay.

The FV stock used in the experiments was a FV complex containing B-tropic F-MuLV and polycythemia-inducing SFFV. The stock was prepared as a 15% spleen cell homogenate from BALB/c mice infected 14 days previously with 3,000 spleen focus-forming units (SFFU). Mice were infected intravenously with 20,000 SFFU for acute infection. For the development of chronic infection, additional 100,000 SFFU of F-MuLV were added. The stock was lactate dehydrogenase virus (LDV)-free.

### Expression of Type I IFN and Determination of IFN Concentrations

Expression of IFNβ were performed as previously described (29). To produce murine IFNα11, the cell line HEK293mIFNalpha11 was cultivated as described (30). All concentrated supernatants were tested for type I IFN activity using the murine 3T3 ISRE Luc reporter cell line, transfected with a plasmid containing the *Firefly Luciferase* gene, stably integrated under control of the IFN-stimulation-response element (ISRE). After 4.5h of stimulation with IFNα, cells were lysed and chemiluminescence was detected using the Beetle-Juice Luciferase assay Firefly (PJK). The IFN activity was calculated to the respective activity in units against commercially available recombinant mouse IFNβ and universal IFNα (PBL assay science).

### *In Vitro* F-MuLV Inhibition Assay

*Mus dunni* tail fibroblast cells were pre-treated *in vitro* for 24 h with increasing concentrations (10 – 10,000 units/ml) of IFNα11 or IFNβ. Cells were then infected with 250 FFU of F-MuLV, cultivated for 3 days, fixed with ethanol, stained with F-MuLV envelope-specific monoclonal antibody 720, and developed with peroxidase-conjugated goat anti-mouse antibody and aminoethylcarbazol to detect viral foci (31).

### IFNα Subtype Treatment *In Vivo*

Mice were injected intraperitoneally daily from days 5 to 9 during acute FV infection or days 40 to 44 of chronic FV infection with 8000 units of IFNα11 or IFNβ. Mock-treated control mice were injected with the supernatant of HEK293T cells transfected with an empty vector. Ten days or 45 post infection, the mice were sacrificed and analyzed for viral loads and immune responses.

### Detection of Virus-Infected Cells

Infectious center (IC) assays were performed on *Mus dunni* tail fibroblast cells as described previously (31). Briefly, titrations (10^7^-10^2^ cells/mL) of single-cell suspensions from infected mouse spleens were plated onto susceptible *M. dunni* cells, co-cultivated for 3 days, and stained with F-MuLV envelope-specific monoclonal antibody 720 to detect foci.

### Cell Surface and Intracellular Staining by Flow Cytometry

Cell surface and intracellular staining of spleen cells was performed as previously described (32, 33) using the following antibodies (BioLegend): anti-CD4 (GK1.5), anti-CD8 (53-6.7), anti-CD43 (1B11), anti-CD62L (MEL-14), anti-Granzyme B (GzmB; clone GB11), anti-IFNγ (XMG1.2), anti-IL-2 (JES6-5H4) and anti-TNFα (MP6-XT22). For intracellular staining, the cells were treated with 10 μg/mL immobilized αCD3 (145-2C11), 2 μg/mL αCD28 (37.51) and 2 μg/mL Brefeldin A in RPMI medium (complemented with 50 μM β-mercaptoethanol) at 37°C for 5 h. Dead cells were excluded from analysis (positive for fixable viability dye, Thermo Scientific). Fluorescence minus one (FMO) controls were used for all conditions. Data were acquired on a FACS LSR II flow cytometer (BD Biosciences) and analyses were performed using Flow Jo (BD Biosciences) software.

### RNA Isolation

Total RNA was isolated from splenocytes utilizing Quick RNA Miniprep (Zymo Research). Isolated RNA was dissolved in RNase-free water and stored at -80°C.

### Real-Time-PCR

Real-time-PCR (RT-PCR) analysis for the quantification of *Oas1a*, *Pkr* and *Isg15* mRNA was performed using PowrUp™ SYBR^®^ Green Master Mix (Thermo Scientific) and QuantiTect Primer Assays (Qiagen) for all 3 genes. The quantitative mRNA levels were determined by using StepOne Software v2.3 (Thermo Scientific) and were normalized to *β-actin* mRNA (Primer forward: caagaaggaaggctggaaaa; Primer reverse: aaatcgtgcgtgacatcaaa) expression levels.

### IFNα Detection in Serum of FV-Infected Mice

The levels of IFNα in the serum of FV-infected mice were detected by using LumiKine™ Xpress mIFN-α 2.0 (*In vivo*gen) according to the manufacturer’s instructions.

To further determine IFNα and IFNβ levels in serum of treated mice, NIH 3T3 cells were seeded in 48-well plates in 10% DMEM and grown under standard cell culture conditions until 70% confluency. The ISRE-Luc reporter plasmid was prepared with polyethylenimine (PEI) in DMEM without FCS and incubated for 20 min at room temperature. Subsequently, the culture medium of the seeded cells was replaced by 500 µL of DMEM supplemented with 2% FCS before applying equal amounts of the transfection mixture. The cells were incubated 16 h at 37°C before transfection. Afterwards, the cells were washed once and stimulated with mice serum (diluted 1:5) for 6 h at 37°C. Then, cells were lysed and chemiluminescence was detected by the Beetle-Juice Luciferase assay Firefly (PJK).

### F-MuLV-Neutralizing Antibody Assay

For analysis of neutralizing antibodies, plasma samples were inactivated for 30 minutes at 56°C and serially diluted with PBS. Plasma dilutions were mixed with purified F-MuLV and guinea pig complement (Sigma-Aldrich) and incubated for 1 h at 37°C. Afterwards, the samples were added to *Mus dunni* cells which were plated in 24-well plates the day before. Cells were incubated to ~100% confluency under standard tissue culture conditions and fixed and stained as described for the IFNα inhibition assay. Foci were counted and dilutions which resulted in at least 75% reduction of foci number were considered neutralizing.

### *In Vivo* Cytotoxicity Assay

For the *in vivo* cytotoxicity assays, 2 x 10^6^ splenocytes loaded with a peptide derived from the FV Gag protein (sequences: CCLCLTVFL) CellTrace™ Violet^high^ (80µM) labelled and 2 x 10^6^ unloaded CellTrace™ Violet^low^ (2µM) labelled splenocytes from naive C57BL/6 mice were adoptively transferred into chronically FV-infected and IFN-treated mice (32). Naive C57BL/6 recipient mice were used as controls to calculate the elimination of target cells. Two hours post transfer, recipient mice were sacrificed and cells were stained with fixable viability dye. The percentage of target-specific killing was calculated as follows: 100 - ([(% peptide pulsed CellTrace™ Violet^hi^ cells in adoptively transferred mice/% unpulsed CellTrace™ Violet^lo^ cells in adoptively transferred mice)/(% peptide pulsed CellTrace™ Violet^hi^ cells in naive/% unpulsed CellTrace™ Violet^lo^ cells in naive)] x 100).

### Statistical Analysis

Experimental data were reported as means +SEM. Statistically significant differences between the IFNα-treated groups and the untreated group were analyzed using Kruskal-Wallis one-way or Ordinary One-Way ANOVA analysis with Dunn’s or Tukey´s multiple comparison *post hoc* test. Statistical analyses were performed using GraphPad Prism software (GraphPad).

## Results

### Type I IFNs Inhibit Acute FV Infection *In Vivo*

**Figure 1:**
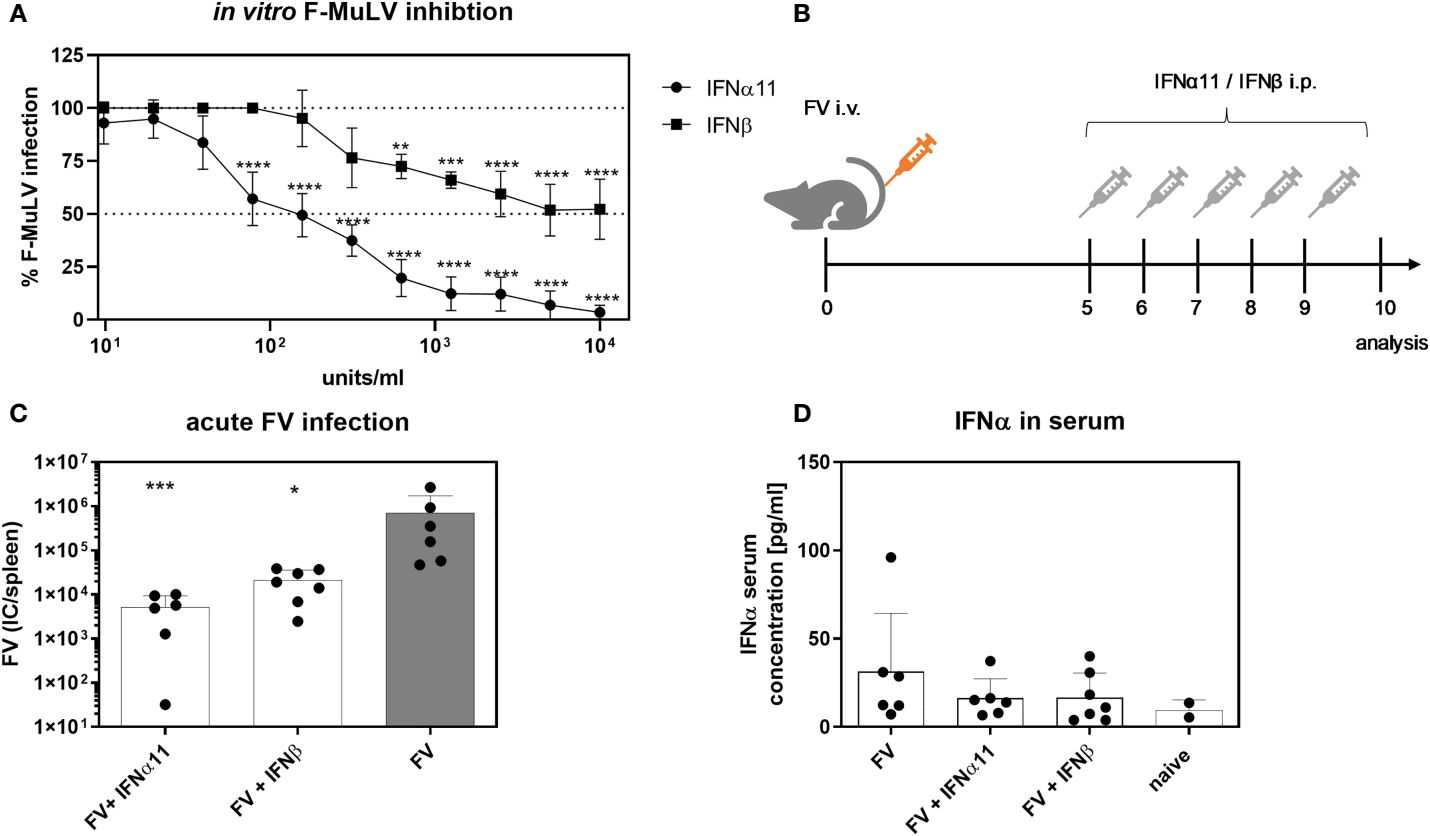
Antiretroviral activity of IFNα11 and IFNβ during acute FV infection. **(A)** Antiretroviral activity of IFNα11 and IFNβ *in vitro*. *Mus dunni* cells were treated *in vitro* with increasing concentrations of IFNα11 and IFNβ (9.76 -10,000 units/ml). Cells were infected with 250 FFU/ml of F-MuLV, cultivated for 3 days, fixed with ethanol, stained with F-MuLV envelope-specific antibody 720 and foci were counted. F-MuLV titers were normalized to untreated controls as % infection and are shown as mean +SEM (n=4). Statistically significant differences between the unstimulated cells (100% infection) and the groups of IFN-stimulated cells (IFNα11 or IFNβ) were tested using Two-way ANOVA and Sidak multiple comparison and are indicated by ** for p < 0.01, *** for p < 0.001, **** for p < 0.0001. **(B)** The scheme of the experimental timeline is shown. C57BL/6 mice were infected with 20,000 SFFU of FV from day +5 to +9 days post infection were treated daily with 8000 units of IFNα11 or IFNβ. Ten dpi, viral loads were analyzed by an infectious center assay **(C)** and serum IFNα concentrations **(D)** were determined by ELISA. Six mice per FV-infected and IFNα11-treated group and 7 mice per IFNβ-treated group were analyzed and the mean values for each group are indicated by a bar (+SEM). Data were pooled from two independent experiments with similar results. Statistically significant differences between the control group (FV) and the groups of IFN-treated mice (FV + IFNα11 or FV + IFNβ) were tested using Kruskal-Wallis one-way and Dunn’s multiple comparison and are indicated by * for p < 0.05, *** for p < 0.001.

Type I IFNs are able to efficiently inhibit acute viral replication. It was already shown, that during acute LCMV infection type I IFNs contribute to the control of viral infection, whereas during chronic LCMV infection, IFNβ, in contrast to IFNα, has a rather detrimental role and contributed to immune dysfunction during persistent LCMV infection. As the role of type I IFNs during retroviral infections is still controversially discussed, we wanted to analyze the specific antiviral activity of IFNα and IFNβ during acute and chronic Friend retrovirus infection *in vivo*. Due to its previously shown high antiviral activity in FV infection, we chose IFNα11 as representative for IFNα in our study (27). Both IFNs were produced, purified and tested for their *in vitro* activity against commercially available IFNs using an ISRE Luc reporter cell line (**Supplementary Figure 1**). To determine their antiretroviral potential, we titrated both type I IFNs against F-MuLV helper virus *in vitro*. Both type I IFNs efficiently suppressed F-MuLV replication *in vitro*, however IFNα11 was much more potent (IC_50_: 128.9 U/ml) than IFNβ (IC_50_: 468.4 U/ml) in controlling F-MuLV infection (**Figure 1A**). Next, we infected C57BL/6 mice with FV and therapeutically applied IFNα11 or IFNβ on five consecutive days starting at day 5 post infection (**Figure 1B**). At day 10 post infection, mice were sacrificed and viral loads in the spleen were determined. Both treatments (IFNα11 and IFNβ) resulted in a significant reduction of viral replication compared to FV-infected control mice (134-fold and 33-fold reduction, respectively) (**Figure 1C**). FV infection itself did not induce a systemic IFNα response, only a transient and low increase in *Ifna* mRNA expression in splenocytes 72 hours post FV infection was detected (29). Type I IFNs have a short half-life *in vivo*, so it was not surprising that 24 hours after the last IFN injection, levels of IFNα were similar between treated mice and FV-infected controls (**Figure 1D**). Furthermore, we also analyzed the serum concentrations of IFNα using an ELISA and both IFNs using a cell-based luciferase assay shortly after injection of the IFNs. ISRE-dependent luciferase activity was detected in all mice receiving IFNα11 and IFNβ after 30, 90, and 240 min post infection to similar extent (**Supplementary Figure 2**). Thus, there were no big differences in IFN bioavailability between the groups.

### IFNα11, But Not IFNβ, Efficiently Controlled Chronic FV Infection

**Figure 2:**
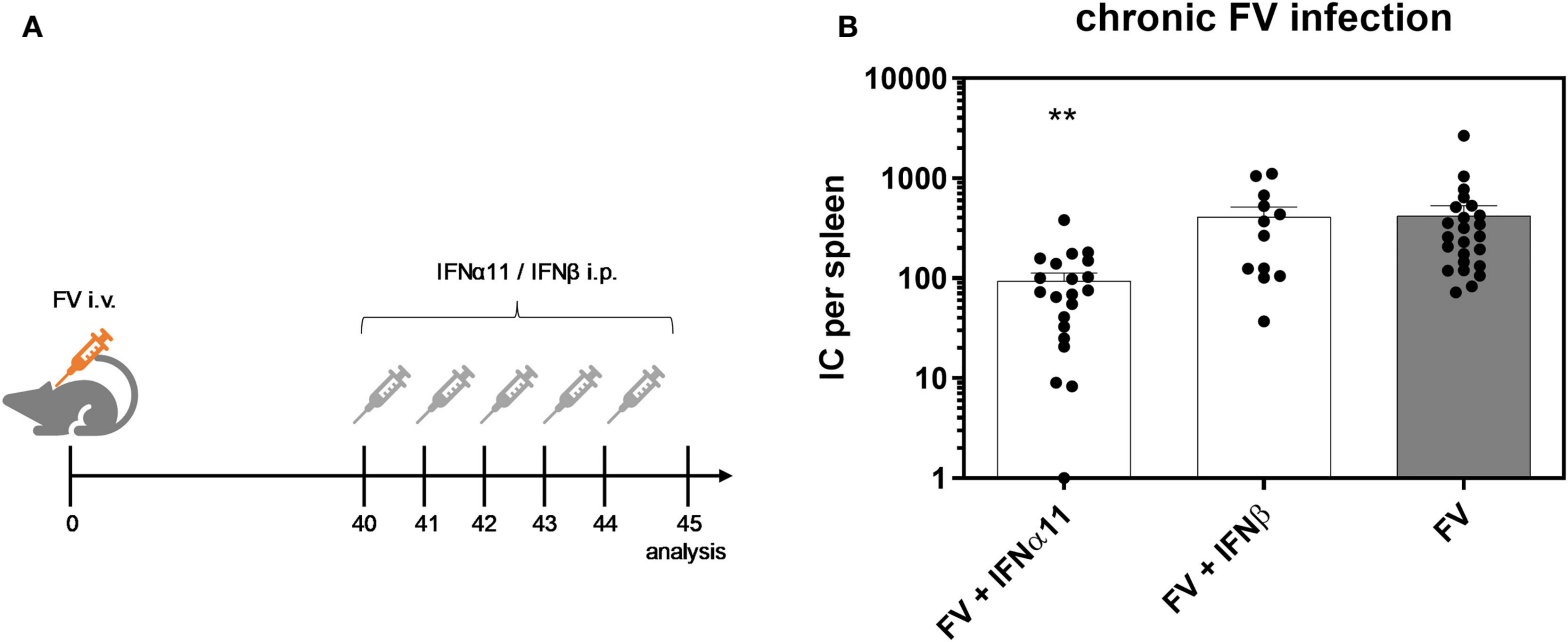
Antiretroviral activity of IFNα11 and IFNβ during chronic FV infection. **(A)** The scheme of the experimental timeline is shown. C57BL/6 mice were infected with 20,000 SFFU of FV and additional 100,000 FFU of F-MuLV. Mice were treated daily with 8000 units of IFNα11 or IFNβ from day 40 to 44 mice. At day 45 post infection, mice were sacrificed and viral loads were analyzed by an infectious center assay **(B)**. 24 mice per FV-infected group, 21 mice per IFNα11-treated group and 12 mice per IFNβ-treated group were analyzed and the mean values for each group are indicated by a bar (+SEM). Data were pooled from four independent experiments with similar results. Statistically significant differences between the control group (FV) and the groups of IFN-treated mice (FV + IFNα11 or FV + IFNβ) were tested using Kruskal-Wallis one-way and Dunn’s multiple comparison and are indicated by ** for p < 0.01.

**Figure 3:**
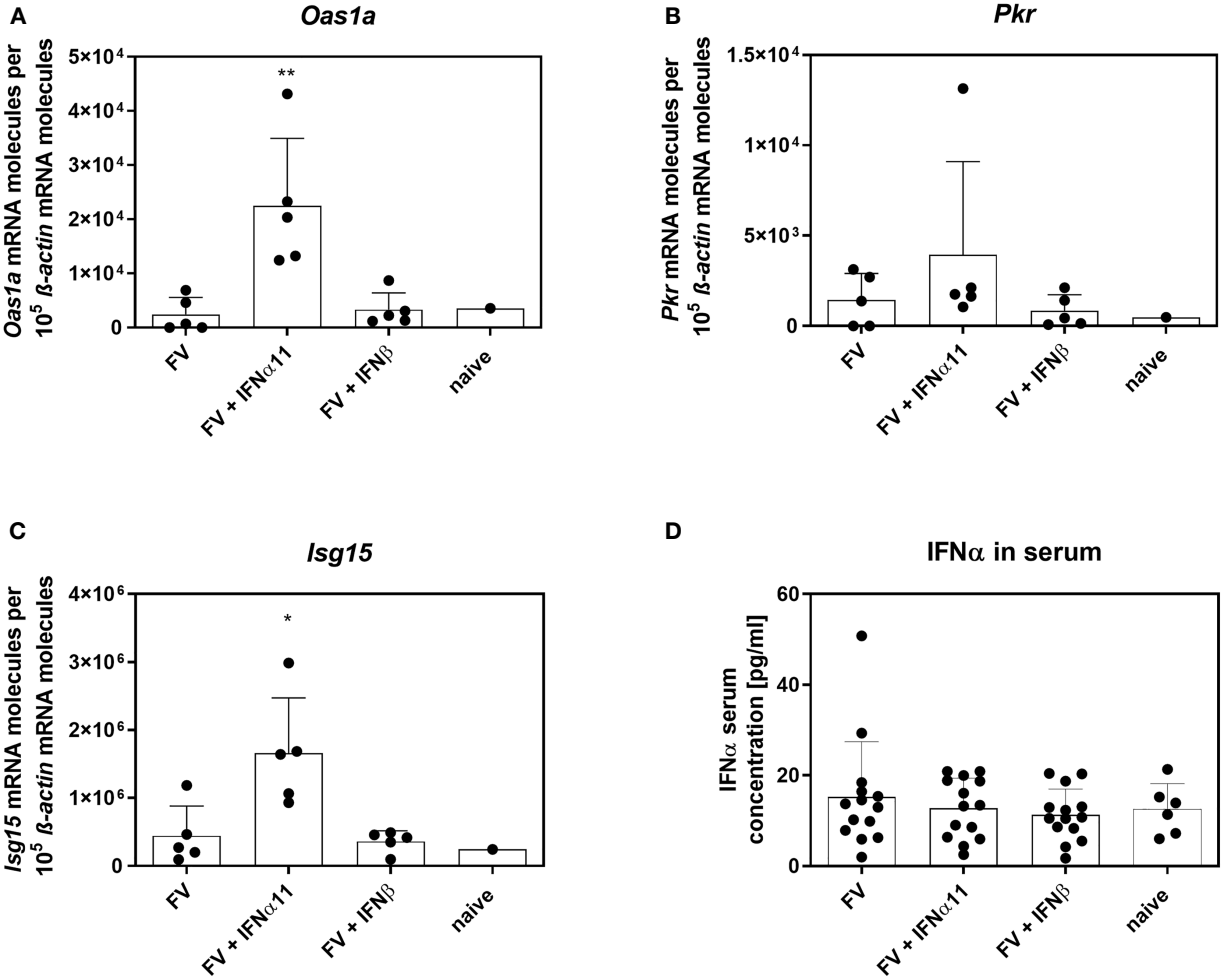
IFN signature in chronically FV-infected mice. C57BL/6 mice were infected with 20,000 SFFU of FV and additional 100,000 FFU of F-MuLV. Mice were treated daily with 8000 units of IFNα11 or IFNβ from day 40 to 44 mice. At day 45 post infection, mice were sacrificed and splenocytes were analyzed for *ISG* mRNA expression **(A–C)**. IFNα serum concentrations of chronically FV-infected mice were determined by ELISA at 45 dpi **(D)**. Five mice per group **(A–C)** and 14 mice per group pooled from three independent experiments **(D)** were analyzed and the mean values for each group are indicated by a bar (+SEM). Statistically significant differences between the control group (FV) and the groups of IFN-treated mice (FV + IFNα11 or FV + IFNβ) were tested using Kruskal-Wallis one-way and Dunn’s multiple comparison and are indicated by *p < 0.05, **p < 0.01.

To elucidate the antiviral efficacy of type I IFNs during chronic FV infection, we infected C57BL/6 mice with FV and let chronicity develop. At day 40 post infection we started the treatment with IFNα11 or IFNβ on five consecutive days. At day 45 post infection, mice were sacrificed and spleens were analyzed for viral titers (**Figure 2A**). During acute FV infection, viral loads peak at 7dpi and further decreased steadily until a persistent low-level infection is established due to efficient CD8 effector T cell responses during acute FV infection (32). Therapeutic application of five doses of IFNα11 during chronic FV infection led to a significant reduction in viral loads (mean viral loads per spleen: 93), whereas treatment with IFNβ did not change viral titers (mean viral loads per spleen: 410 IC in IFNβ-treated mice and 422 in untreated controls) (**Figure 2B**). Furthermore, we monitored the IFN signature during chronic FV infection by analyzing the expression of selected ISGs as well as the concentration of IFNα in the serum of chronically FV-infected mice. As shown in **Figures 3A**–**C**, chronic FV infection did not induce a significant expression of ISGs (*Oas1a*, *Pkr*, *Isg15*) in splenocytes compared to uninfected control mice. In contrast, treatment with IFNα11 significantly induced the mRNA expression of some ISGs (*Oas1a*, *Isg15*). Surprisingly, therapy with IFNβ did not alter the expression of the studied ISGs, indicating that only IFNα11, but not IFNβ, induced a significant ISG response during chronic FV infection. Similar to acute FV infection, the virus itself did not induce a systemic IFNα responses, and also 24 hours post IFN-treatment no increased IFNα serum concentrations were found (**Figure 3D**). These data imply, that in contrast to acute FV infection, IFNβ could not control chronic FV infection. However, IFNα11 was able to suppress FV replication during acute and chronic infection.

### Modest Immunomodulatory Effects of Type I IFNs on CD4^+^ T Cells

**Figure 4:**
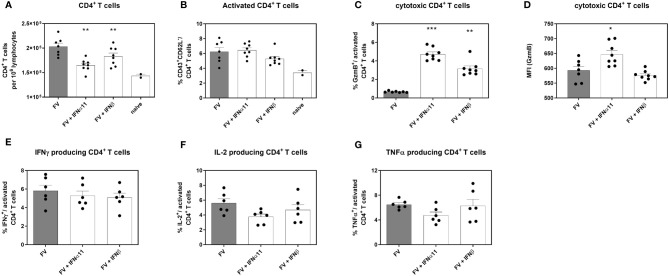
Analysis of intracellular cytotoxic molecules and cytokine expression of CD4^+^ T cells in type I IFN-treated chronically FV-infected mice. C57BL/6 mice were infected with 20,000 SFFU of FV and additional 100,000 FFU of F-MuLV. Mice were treated daily with 8000 units of IFNα11 or IFNβ from day 40 to 44 mice. At day 45 post infection, mice were sacrificed and CD4^+^ T cell effector functions were analyzed by flow cytometry. Numbers of CD4^+^ T cells **(A)**, percentages of CD43^+^ CD62L^-^activated CD4^+^ T cells **(B)**, percentages of intracellular expression determine of **(C)** GzmB and **(D)** individual GzmB expression (MFI) was determined. Multi-parametric flow cytometry was used to measure percentages of intracellular expression of IFNγ **(E)**, IL-2 **(F)** and TNFα **(G)** in activated CD4^+^ T cells. Mean values (+SEM) are indicated by bars. Statistically significant differences between the IFN-treated groups and the untreated group were analyzed using Kruskal-Wallis one-way analysis and Dunn’s multiple comparison and are indicated by * for p < 0.05; ** for p < 0.01; *** for p < 0.001.

Type I IFNs are defined by their antiviral properties, but they are also potent immunomodulators that can act directly on different cells of the innate and adaptive immune systems. Type I IFNs can modulate the activation, effector function, and survival of T cells (34–37). To clarify the biological activity of type I IFNs during chronic FV infection, we analyzed the influence of type I IFNs on CD4^+^ T helper cells during chronic FV infection. CD4^+^ T cells have no direct antiviral activity during acute FV infection; however, they are required for the control of chronic infection by mediating direct antiviral effects (38, 39). We observed a significant decrease in the numbers of CD4^+^ T cells in the spleen after treatment with both type I IFNs compared to untreated controls, but the numbers were still higher than in uninfected mice (**Figure 4A**). These results nicely confirm previous *in vitro* data, in which different IFNα subtypes reduced the proliferation of FV-specific T cells (37). Treatment with IFNα11 or IFNβ neither changed the frequencies of activated CD4^+^ T cells (**Figure 4B**), nor the frequencies of cytokine-producing CD4^+^ T cells (**Figures 4E–G**) in comparison to untreated FV-infected control mice. However, IFNα- and IFNβ-treatment significantly enhanced the percentages of cytotoxic CD4^+^ T cells, shown by the intracellular expression of GzmB as well as the individual GzmB expression per cell shown by mean fluorescence intensity (MFI) in activated CD4^+^ T cells (**Figures 4C, D**).

Apart from these cytotoxic CD4^+^ T cells, neutralizing antibodies are also important to keep viral replication in check (40–42). Furthermore, type I IFNs are able to enhance antibody responses (43–45). Thus, we determined the influence of type I IFNs treatment on neutralizing antibody responses. Therefore, serum samples of chronically FV-infected mice and IFN-treated mice were analyzed for their neutralizing capacity. We observed only a slight increase in neutralizing antibody titers against F-MuLV after IFNβ therapy, whereas treatment with IFNα did not affect neutralizing antibody titers (**Supplementary Figure 3**). In conclusion, we found only modest immunomodulatory effects of IFNα11 and IFNβ on CD4^+^ T cell and antibody responses, except for cytotoxic T cell responses.

### Dysfunctional CD8^+^ T Cells Regain Cytotoxic Activity Upon IFNα11 Treatment During Chronic FV Infection

**Figure 5:**
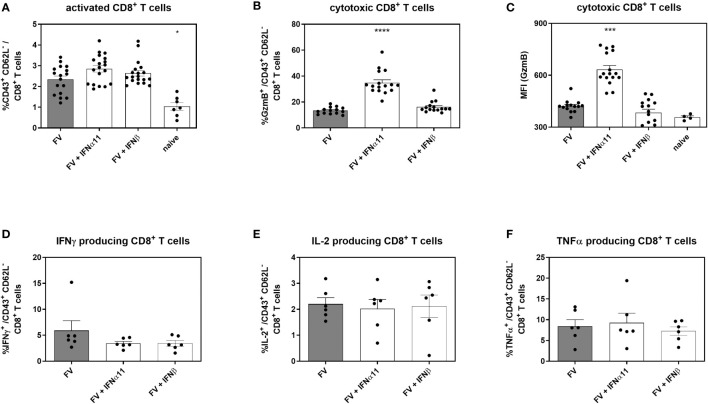
Analysis of intracellular cytotoxic molecules and cytokine expression of CD8^+^ T cells in type I IFN-treated chronically FV-infected mice. C57BL/6 mice were infected with 20,000 SFFU of FV and additional 100,000 FFU of F-MuLV. Mice were treated daily with 8000 units of IFNα11 or IFNβ from day 40 to 44 mice. At day 45 post infection, mice were sacrificed and CD8^+^ T cell effector functions were analyzed by flow cytometry. Percentages of CD43^+^ CD62L^-^ activated CD8^+^ T cells **(A)** and percentages of intracellular GzmB expression **(B)** and individual GzmB expression (MFI) was determined **(C)**. Multi-parametric flow cytometry was used to measure percentages of intracellular expression of IFNγ **(D)**, IL-2 **(E)** and TNFα **(F)** in activated CD8^+^ T cells. Mean values (+SEM) are indicated by bars. Statistically significant differences between the IFN-treated groups and the untreated group were analyzed using Kruskal-Wallis one-way analysis and Dunn’s multiple comparison and are indicated by * for p < 0.05; *** for p < 0.001; **** for p < 0.0001.

**Figure 6:**
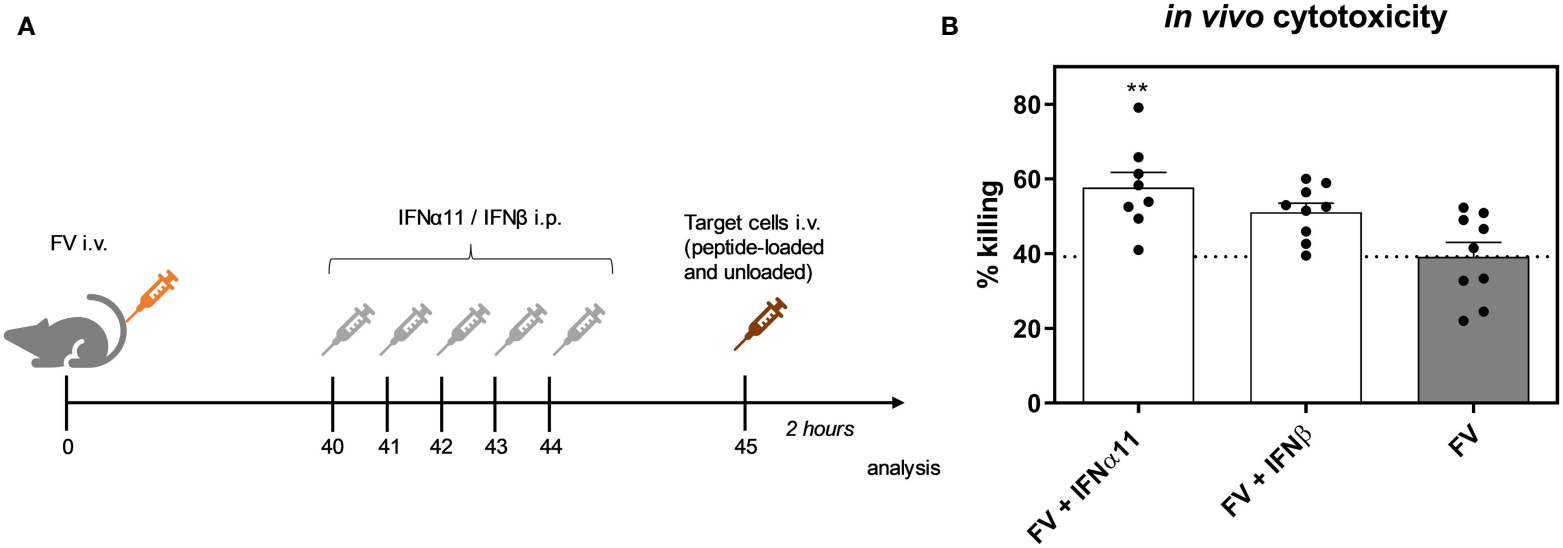
Influence of IFN treatment on the cytotoxic activity of FV-specific CD8^+^ T cells *in vivo*. C57BL/6 mice were infected with 20,000 SFFU of FV and additional 100,000 FFU of F-MuLV. Mice were treated daily with 8000 units of IFNα11 or IFNβ from day 40 to 44 mice. At day 45 post infection, peptide-loaded and Cell Trace™ Violet-labeled target cells (80 µM, high) were mixed with unloaded and Cell Trace™ Violet-labeled target cells (2 µM, low) in a ratio of 1:1 and were injected i.v. into FV-infected and IFN-treated mice. After 2 hours, mice were sacrificed and the killing capacity was determined. **(A)** The scheme of the experimental timeline is shown. **(B)** The percentages (+SEM) of target cell killing in spleen is shown. Statistically significant differences between the groups were tested using ordinary one way ANOVA and Tukey’s multiple comparison and are indicated by ** for p < 0.01.

Cytotoxic CD8^+^ T cells are very effective in restricting viral spread during acute FV infection. They become exhausted by regulatory T cells and *via* the expression of inhibitory receptors during the transition phase between acute and chronic FV infection, leading to dysfunctional CD8^+^ T cells during persistent FV infection. Checkpoint blockade or depletion of regulatory T cells during chronic FV infection reactivates cytotoxic CD8^+^ T cells which then efficiently control persistent FV infection (46, 47). Type I IFNs can enhance the cytotoxicity of CD8^+^ T cells (34), but they can also increase the expression of inhibitory receptors and ligands like PD-1 or PD-L1 (48). Thus, we determined the effect of type I IFN treatment on CD8^+^ T cells during chronic FV infection as shown in **Figure 2A**. In accordance with previously published data (32) the percentages of CD43^+^ CD62L^-^ activated CD8^+^ T cells were increased during persistent FV infection compared to naïve mice (**Figure 5A**). The application of IFNα11 or IFNβ further increased the frequencies of activated CD8^+^ T cells. Interestingly, frequencies of GzmB-expressing activated CD8^+^ T cells as well as the expression levels of GzmB in activated CD8^+^ T cells were strongly enhanced after IFNα11 therapy, whereas the treatment with IFNβ had no effect on the expression of cytotoxic molecules during persistent FV infection (**Figures 5B**, **C**). In line with the CD4^+^ T cell data (**Figures 4E**–**G**), we did not observe any significant changes in the percentages of cytokine producing CD8^+^ T cells (**Figures 5D**–**F**) after type I IFN treatment. To verify that the increased GzmB expression also implicates higher cytotoxicity in the reactivated CD8^+^ T cells, we analyzed their potency to eliminate target cells *in vivo*. Therefore, we treated chronic FV-infected mice with either IFNα11 or IFNβ and at day 45 post infection, we adoptively transferred target cells loaded with an immunodominant epitope peptide derived from the FV Gag protein as well as unloaded cells as control. After two hours of incubation, killing of target cells by FV-specific CD8^+^ T cells was determined in the spleen (**Figure 6A**). Depending on the numbers of transferred target cells and the incubation time, we observed an elimination of 39.2% ± 11.48 of transferred target cells in persistent FV infected mice (**Figure 6B**). We assessed a significant increase in the killing capacity of FV-specific CD8^+^ T cells after treatment with IFNα11 (57.6% ± 11.51). We also observed a slight increase in target-cell killing after IFNβ treatment (51.1% ± 7.1), however this was not statistically significant.

In conclusion, we observed an antiviral effect of IFNα11 and IFNβ during acute FV infection. In contrast, during chronic FV infection, only IFNα11 therapy was able to control FV replication. Apart from the likely direct antiviral activity of IFNα11 suggested by the induction of ISGs, treatment with IFNα11 reactivated dysfunctional CD8^+^ T cells, and augmented their cytotoxic effector function.

## Discussion

Although type I IFNs were shown to be antiviral in different acute viral infections, its role in persistent viral infections is controversially discussed. In this work, we could show that type I IFNs including IFNα and IFNβ controlled acute FV infection, whereas a therapeutic treatment during chronic FV infection was only beneficial after administration of IFNα (11). Interestingly, in contrast to its antiviral effect *in vitro* and in acute FV infection, application of recombinant IFNβ did not control persistent FV infection. Type I IFNs consist of numerous IFNα subtypes, IFNβ, IFNϵ, IFNκ and IFNζ/limitin with broad pleiotropic biological effects including antiviral, antiproliferative, immunomodulatory, and regulatory properties. There are more and more publications showing an either beneficial or detrimental effect of type I IFNs for the host which depends on the pathogen, pathogen load, the timing, the infected cell type, and the type of IFN-producing cell. Careful detailed investigations of the unique properties of type I IFNs in different infection models are required to get a better understanding of type I IFN-mediated responses and their underlying mechanisms.

During chronic viral infections hyperimmune-activation, the expression of negative immune regulators (IL-10 and PD-L1), an increased IFN signature, and destruction of the lymphoid tissue architecture correlate with disease progression in LCMV, HIV/SIV, or HCV infection (24, 49–51). Here, we did not detect an elevated IFN signature in the chronically FV-infected mice neither by systemic IFNα levels nor by mRNA expression of some key ISGs. The overall IFN response in untreated persistent FV-infected animals was comparable to the basal expression levels in uninfected controls. An induction of the immunoregulatory ligand PD-L1 was shown to increase on virus-infected cells during acute FV infection and that the expression of PD-L1 could be further increased upon type I IFN stimulation *in vitro* (52). However, during chronic FV infection the expression of PD-L1 is comparable to baseline expression levels in naive mice (data not shown). Thus, during chronic Friend retroviral infection, the mice did not develop chronic hyperimmune-activation, elevated IFN signatures, or upregulated negative immune regulators. The host immune response during chronic FV infection is characterized by exhausted FV-specific CD8^+^ T cells which can be reactivated through Treg depletion or α-PD-L1 antibody treatment (32, 46). Here, we could show that IFNα11 treatment in chronic FV infection, induced the expression of antiviral ISGs, but also stimulated exhausted CD8^+^ T cells that regained effector cell function.

To uncover the different biological roles of type I IFNs their impact on modulating host immune responses has to be identified in detail. In our study the therapeutic treatment with IFNα11 resulted in an increased cytotoxicity of CD8^+^ T cells shown by the expression of granzyme B and an improved target cell killing *in vivo*. In contrast, IFNβ did not increase the expression of granzyme B, but a trend to slight increase in target cell killing was observed which was not as strong as after IFNα11 treatment. Various immunomodulatory roles of type I IFNs were already described like activation of DCs, increased NK cell cytotoxicity, improved T cell effector functions, and activation of B cells (27, 36, 37, 43, 44, 53). In the current study, only differences in T cell responses were detected, as neutralizing antibody titers only slightly improved after IFNβ treatment in chronic FV infection and might therefore play a minor role in the beneficial outcome of the IFN-immunotherapy. In chronic LCMV infection IFNα was shown to control early viral dissemination, but it does not affect persistent viral infection (24). Interestingly, blocking of IFNβ but not IFNα improved antiviral T cell responses and reduced viral loads by decreasing the amounts of infected CD8α^-^ DCs and preventing disruption of the splenic architecture. In a previous study the authors also showed increased levels of the negative immunoregulators IL-10 and PD-L1 in αIFNAR-treated LCMV-infected mice (22). However, IFNβ blockade during persistent LCMV infection did not result in a reduction of PD-L1 expression of antigen-presenting cells or serum IL-10 level (24). We also did not detect any significant differences in PD-1/PD-L1 expression or systemic IL-10 levels after IFNβ therapy in chronic FV-infected mice (data not shown), which might explain the diverse activity of these two type I IFNs in persistent FV infection. We might speculate that the reduced direct antiviral activity by IFNβ (shown by no increase in ISG expression, **Figure 3**), and the slight effect on T cell cytotoxicity (**Figures 4C**, **6B**) accounted for the significant therapeutic difference of IFNβ versus IFNα11 during chronic FV infection. However, this only accounted for chronic FV infection, as IFNβ therapy significantly reduced viral loads during acute FV infection. Another study in SIV-infected macaques describes the importance of timing and duration of an IFN-immunotherapy (54). Application of IFNα2a initially upregulated the expression of antiviral genes and prevented a systemic SIV-infection. Longer treatments resulted in desensitization of type I IFNs and reduced ISG expression leading to an increased SIV reservoir size. This might account for all the different members of the type I IFN family as their unique biological activity might depend on infecting pathogen, infected tissue/cell type and the phase of the infection. The use of IFNα in the treatment of HIV-1 infection or as a cure strategy is controversial, particularly due to several studies blocking the IFNα/β receptor in HIV-1 infected humanized mice. One study using monoclonal antibodies to block IFNAR during persistent HIV infection demonstrated that, despite having increased viral loads upon blockade, IFNAR signaling may drive CD4^+^ T cell apoptosis and dysfunction of CD4^+^ and CD8^+^ T cells during chronic infection in humanized mice (20). Additionally, others reported that antiretroviral therapy combined with IFNAR blockade in HIV-1 infected humanized mice decreased plasma RNA levels as well as numbers of latently infected cells (19). In contrast to these studies that block all type I IFN-mediated effects including IFNα and IFNβ-mediated antiviral and immunomodulatory effects, we and others have reported that specific IFNα subtypes can mediate beneficial effects in HIV-1 infected humanized mice (10, 12, 55).

IFNα2a/b is clinically approved for the treatment against HBV and HCV; however, HCV infection is nowadays treated with direct acting antivirals, which are the safest and most effective medicines for treating hepatitis C with a success rate of more than 90%. Immunotherapy with IFNα2a/b is important for the clinical treatment of chronic hepatitis B. IFNα exhibits direct antiviral effect as well as immunomodulatory activities, which can induce sustained antiviral responses in part of the treated chronic hepatitis B patients. IFNα2a/b therapy inhibits viral replication intermediates, blocks reinfection and improves clearance of infected hepatocytes through stimulation of immune cell responses. IFNα is also able to reduce the covalently closed circular DNA pool of HBV, but the HBsAg clearance rates after IFNα2a/b treatment are rather low (up to 30%). Importantly, up to date only one IFNα subtype is approved for clinical treatment, and the unique and non-redundant antiviral and biological functions of the other eleven human IFNα subtypes are not considered at all. IFNβ is also approved for clinical treatment against multiple sclerosis (MS), but not as an antiviral drug. The underlying molecular mechanism of IFNβ in MS is still elusive, but some reports showed an increased production of anti-inflammatory cytokines, decreased major histocompatibility complex II (MHC II) expression on antigen-presenting cells, a diminished lymphocyte activation, and reduced T cell migration through the blood-brain barrier (56–58). Similar effects were also observed in a mouse model of cerebral malaria in which treatment with IFNβ increased the survival rate of the mice and improved the blood-brain barrier function, but it did not alter the systemic parasitemia of *Plasmodium berghei* (59). These observations clearly describe a more regulatory function of IFNβ, which was also reported elsewhere (24, 60, 61) and which further confirmed our findings of IFNβ in chronic FV infection.

In conclusion, we could show that during persistent FV infection only the treatment with IFNα enables retroviral control, whereas recombinant IFNβ could only control acute FV infection. Our study demonstrates the pleiotropic biological activity of different type I IFNs, although they all bind to the same receptor and activate the same downstream signaling cascades. Further detailed analysis is required to fully understand the complexity of the type I IFN responses in viral infections.

## Data Availability Statement

The raw data supporting the conclusions of this article will be made available by the authors, without undue reservation.

## Ethics Statement

The animal study was reviewed and approved by North Rhine-Westphalia State Agency for Nature, Environment and Consumer Protection (LANUV).

## Author Contributions

KS and UD conceived of the study. MS, YS, AM, ZK, and JD substantially contributed to the acquisition and analysis of the data. TK contributed to the implementation of the research. KS wrote the original manuscript. All authors edited and approved the final manuscript.

## Funding

This work was supported by the DFG RTG 1949 to KS and UD.

## Conflict of Interest

The authors declare that the research was conducted in the absence of any commercial or financial relationships that could be construed as a potential conflict of interest.

## Publisher’s Note

All claims expressed in this article are solely those of the authors and do not necessarily represent those of their affiliated organizations, or those of the publisher, the editors and the reviewers. Any product that may be evaluated in this article, or claim that may be made by its manufacturer, is not guaranteed or endorsed by the publisher.

## Supplementary Material

Supplementary Figure 1 Type I IFN activity on ISRE Luc reporter cells. 3T3 ISRE Luc reporter cells were stimulated with different concentrations of IFNα11 and IFNβ for 4.5h. As control commercially available universal IFNα and IFNβ (PBL) were used. Luciferase activity was determined in relative light units (RLU). Three independent experiments were performed and are shown as individual data sets.

Supplementary Figure 2 Serum type I IFN levels at different timepoints post IFN injection. C57BL/6 mice were treated with 8000 units of IFNα11 or IFNβ. At 30, 90, and 240 min post injection, mice were sacrificed and type I IFN levels were measured in the serum by IFNα-specific ELISA (A) and ISRE-dependent firefly luciferase activity (B). Statistically significant differences between the control group (0) and the groups of IFN-treated mice (IFNα11 or IFNβ) were tested using Kruskal-Wallis one-way and Dunn’s multiple comparison and are indicated by *p < 0.05.

Supplementary Figure 3 Neutralizing antibody titers in chronically FV-infected mice. C57BL/6 mice were infected with 20,000 SFFU of FV and additional 100,000 FFU of F-MuLV. Mice were treated daily with 8000 units of IFNα11 or IFNβ from day 40 to 44 post infection. At day 45 post infection, mice were sacrificed and serum samples were analyzed for neutralizing antibody titers. At least 8 mice per group from two individual experiments were analyzed and the mean values for each group are indicated by a bar (+SEM). Statistically significant differences between the control group (FV) and the groups of IFN-treated mice (FV + IFNα11 or FV + IFNβ) were tested using Kruskal-Wallis one-way and Dunn’s multiple comparison and are indicated by *p < 0.05.
