# Supplementary figures and images for: Immunotherapy With Interferon α11, But Not Interferon Beta, Controls Persistent Retroviral Infection
Source: Front Immunol. 2022 Jan 20;12:809774. doi: 10.3389/fimmu.2021.809774 (PMC8810532; doi:10.3389/fimmu.2021.809774)

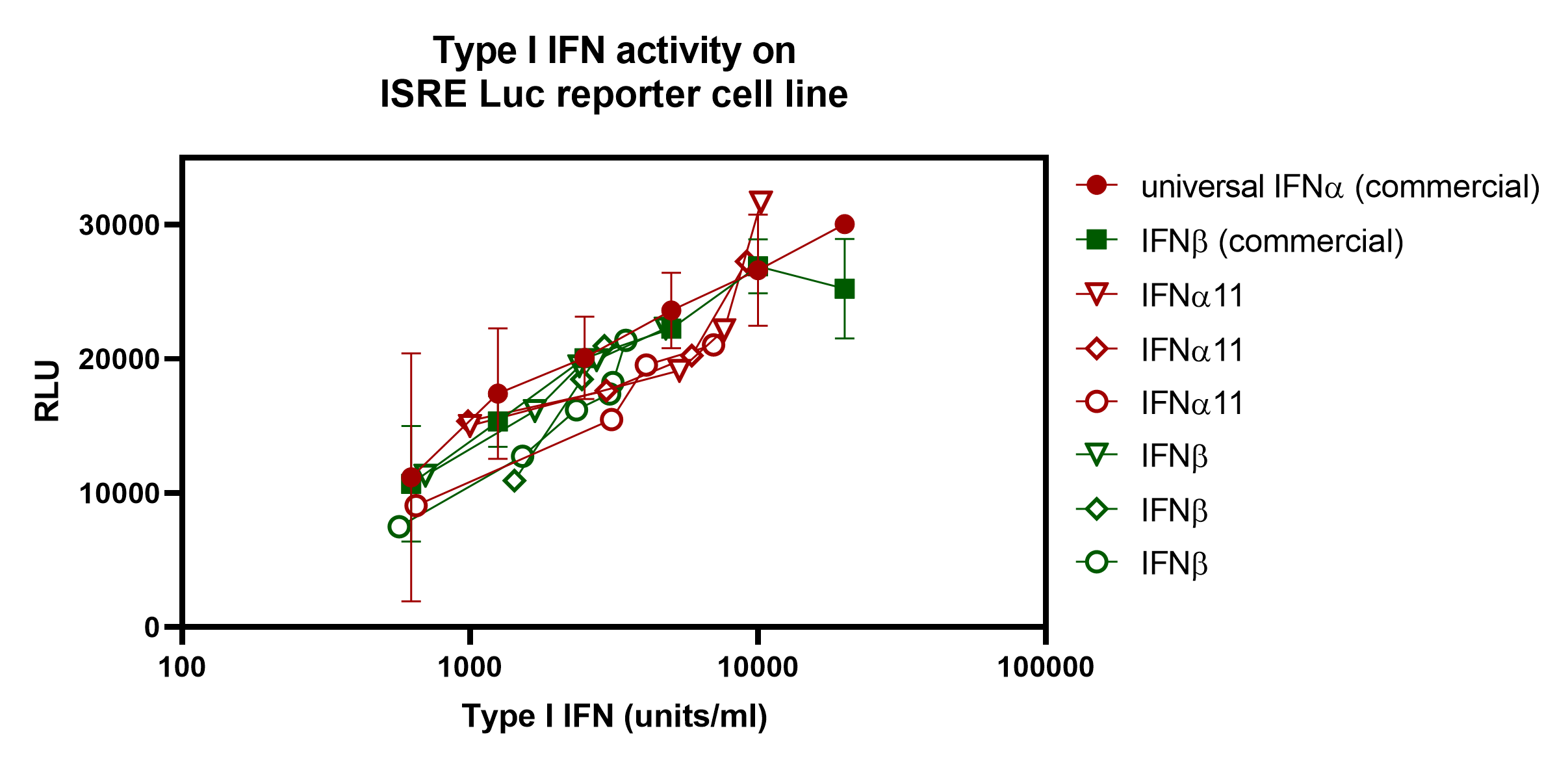

Supplement: Supplementary Figure 1 — Type I IFN activity on ISRE Luc reporter cells. 3T3 ISRE Luc reporter cells were stimulated with different concentrations of IFNα11 and IFNβ for 4.5h. As control commercially available universal IFNα and IFNβ (PBL) were used. Luciferase activity was determined in relative light units (RLU). Three independent experiments were performed and are shown as individual data sets. [file Image_1.tif]

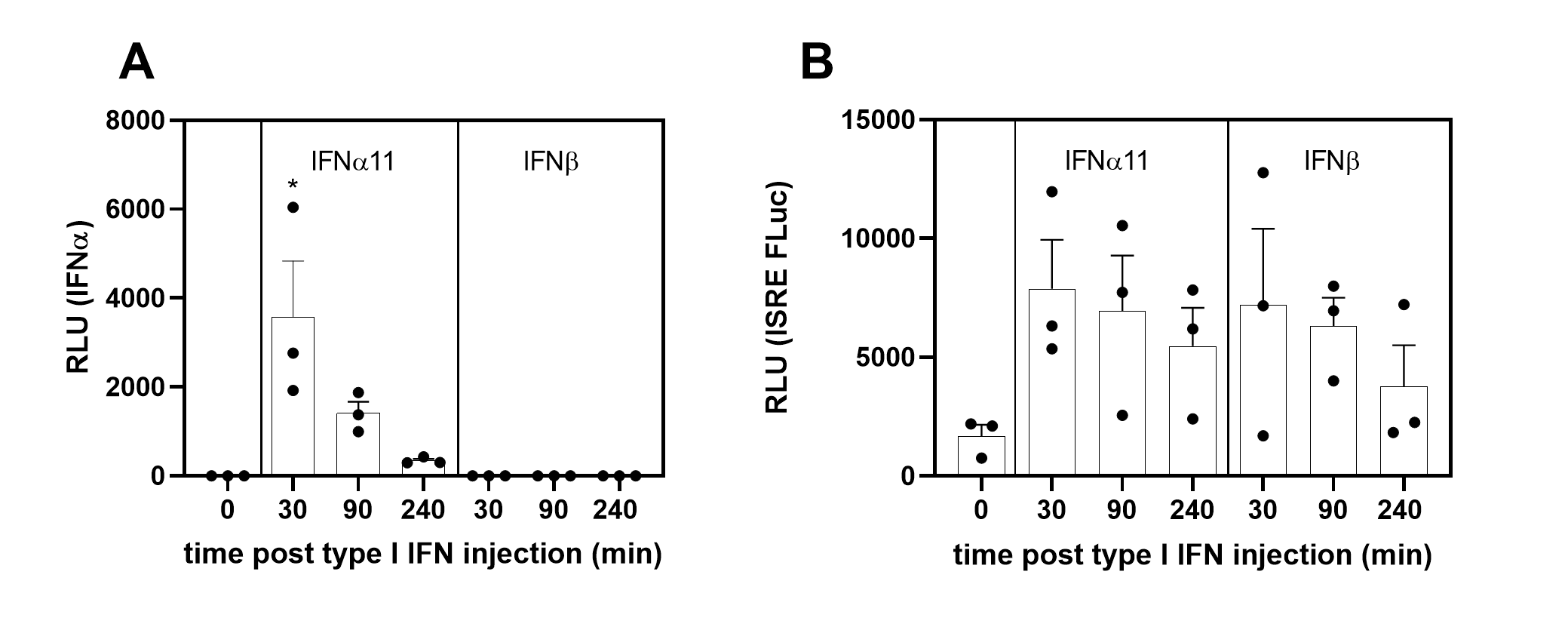

Supplement: Supplementary Figure 2 — Serum type I IFN levels at different timepoints post IFN injection. C57BL/6 mice were treated with 8000 units of IFNα11 or IFNβ. At 30, 90, and 240 min post injection, mice were sacrificed and type I IFN levels were measured in the serum by IFNα-specific ELISA (A) and ISRE-dependent firefly luciferase activity (B). Statistically significant differences between the control group (0) and the groups of IFN-treated mice (IFNα11 or IFNβ) were tested using Kruskal-Wallis one-way and Dunn’s multiple comparison and are indicated by *p < 0.05. [file Image_2.tif]

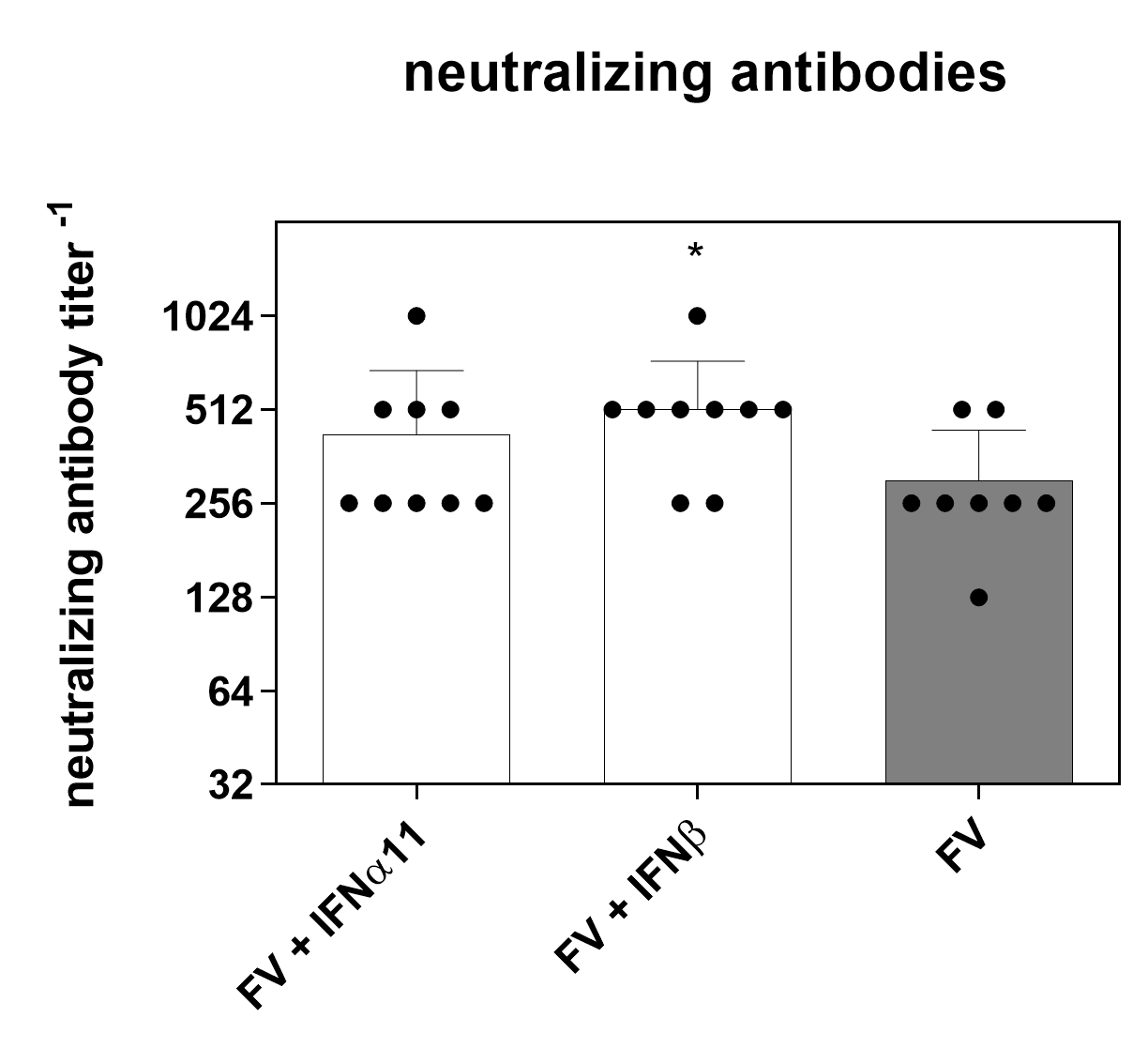

Supplement: Supplementary Figure 3 — Neutralizing antibody titers in chronically FV-infected mice. C57BL/6 mice were infected with 20,000 SFFU of FV and additional 100,000 FFU of F-MuLV. Mice were treated daily with 8000 units of IFNα11 or IFNβ from day 40 to 44 post infection. At day 45 post infection, mice were sacrificed and serum samples were analyzed for neutralizing antibody titers. At least 8 mice per group from two individual experiments were analyzed and the mean values for each group are indicated by a bar (+SEM). Statistically significant differences between the control group (FV) and the groups of IFN-treated mice (FV + IFNα11 or FV + IFNβ) were tested using Kruskal-Wallis one-way and Dunn’s multiple comparison and are indicated by *p < 0.05. [file Image_3.tif]
